# Modulators of Airway Remodeling: The Role of Caffeine and Calcitriol

**DOI:** 10.3390/ijms27073087

**Published:** 2026-03-28

**Authors:** Marharyta Sobczak, Joanna Wieczfińska, Rafał Pawliczak

**Affiliations:** Department of Immunopathology, Division of Biomedical Science, Medical Faculty, Medical University of Lodz, 90-752 Lodz, Poland; marharyta.sobczak@umed.lodz.pl (M.S.); joanna.wieczfinska@umed.lodz.pl (J.W.)

**Keywords:** vitamin D, caffeine, calcitriol, airway remodeling, asthma

## Abstract

Airway remodeling is a process that occurs in chronic obstructive diseases, such as asthma and COPD. It is associated with adverse changes in the structure and function of the airways. An increasing amount of literature points to the potential protective effects of vitamin D and caffeine against inflammation and fibrosis. The aim of the study is to evaluate the effect of calcitriol and caffeine on the expression of genes and proteins associated with airway remodeling. The Calu-3 cell line was treated with TGF-β, calcitriol, and caffeine in different combinations. Subsequently, the expression of VDR, CDH1, VIM, MMP-2, and MMP-9 were examined at the mRNA and protein levels using real-time PCR and Western blot, respectively. One-way analysis of variance was used to determine differences in several groups. Both calcitriol and caffeine were associated with a decrease in the expression of *MMP-2* and *VIM* in TGF-β-treated cells (*p* = 0.01 and *p* = 0.006, respectively). Both compounds also reduced the expression of *MMP-9* in comparison to TGF-β alone (*p* = 0.03), though the changes in MMP-9 protein levels did not reach statistical significance. Calcitriol was associated with a decrease in CDH1 expression at both levels in comparison to TGF-β (*p* < 0.0001 and *p* = 0.02, respectively). A potential synergistic effect was demonstrated for *CDH1* at the mRNA level and for the vitamin D receptor at the protein level. Both vitamin D and caffeine may influence the pathways involved in airway remodeling. Preliminary *in vitro* findings suggest a potential role of these substances for future therapeutic strategies targeting obstructive diseases; however, the observations require confirmation in further *in vivo* studies.

## 1. Introduction

Asthma is a heterogeneous chronic inflammatory disease of the respiratory system, which is characterized by airflow limitation. The disease is manifested by shortness of breath, wheezing, coughing and a feeling of tightness in the chest [1]. Asthma endotypes, such as type 2-high and type 2-low, have been identified to distinguish groups of patients who share similar disease characteristics at the cellular and molecular levels. Asthma and chronic obstructive pulmonary disease (COPD) both lead to airflow limitation, which is largely reversible in asthma but only partially reversible in COPD. Although both conditions involve inflammatory and structural airway changes, the types of infiltrating cells and the patterns of remodeling differ between the two diseases [2].

The airway epithelium plays a key role in asthma’s pathophysiology [3]. In addition, it is an initiator of airway remodeling in asthma [1]. Pathogens, pollutants or allergens can damage airway epithelial cells and trigger the release of epithelial cytokines. Abnormal immune responses in asthmatics lead to recurrent or chronic inflammation and damage to the airway epithelium, which can result in structural changes in the airways [3]. These changes, such as epithelial damage, excessive proliferation of mucosal glands, subepithelial deposition of proteoglycans and collagen in the basement membrane, angiogenesis and increased airway smooth muscle mass, are known as airway remodeling [4]. Moreover, airway epithelial cells act as a starting point for airway remodeling in asthma [3]. A large number of cytokines and chemokines are involved in airway remodeling in asthma. Notably, transforming growth factor beta (TGF-β) is the major mediator of airway remodeling by inducing epithelial–mesenchymal transition [1]. Epithelial–mesenchymal transition (EMT) is a complex cellular process in which epithelial cells lose epithelial markers, such as E-cadherin, and acquire a mesenchymal phenotype by activating mesenchymal genes, such as vimentin. This process enables the cells to produce the extracellular matrix (ECM), a key component of airway remodeling [5]. Extracellular matrix metalloproteinases (MMPs) are a group of zinc-dependent enzymes responsible for remodeling ECM components [6].

Coffee is one of the most popular drinks consumed worldwide, and it was recommended in 1859 as one of the best remedies for asthma. Caffeine, which belongs to the methylxanthine group, is found not only in coffee but also in tea and cocoa products [7]. Caffeine is known for its anti-inflammatory effects [8]. Both coffee and caffeine reduced airway hyperresponsiveness in mice [9]. A systematic review of fifteen studies found that coffee consumption was generally associated with a reduction in the incidence of asthma, suggesting a potential protective effect of coffee against asthma [10]. Similarly, a meta-analysis found that caffeine can improve lung function compared to placebo [7]. However, a cross-sectional study revealed that coffee and caffeine consumption were positively associated with an increased risk of COPD [11]. The frequency of COPD exacerbations is not significantly affected by caffeine, according to a retrospective analysis [12]. Therefore, caffeine may protect against asthma and improve lung function, but its effect on the risk and exacerbation of COPD remains unclear. Another well-known immunomodulator that has been studied in relation to asthma is vitamin D. Studies suggest that vitamin D deficiency is associated with more severe disease symptoms. It is also associated with worse lung function. The systematic review, which included *in vitro* studies, indicates that vitamin D has beneficial effects on the airways. These effects include inhibiting airway smooth muscle cell remodeling, reducing inflammation, and regulating collagen synthesis [13]. According to a meta-analysis of randomized controlled trials (RCTs), vitamin D supplementation leads to a reduction in the frequency of moderate and severe COPD exacerbations in individuals with low baseline 25-hydroxyvitamin D concentrations (<25 nmol/L), whereas this effect is not observed in patients with values above this threshold [14].

Although vitamin D is associated with the regulation of the epithelial barrier and caffeine is associated with regulating anti-inflammatory pathways, their combined effect on structural changes in airways and remodeling processes has not been evaluated. Therefore, we decided to test the effect of caffeine and calcitriol, the active metabolite of vitamin D, on TGF-β-induced airway remodeling.

## 2. Results

### 2.1. The Influence of Caffeine and Calcitriol on Expression of Genes Related to Cytoarchitecture and Cell Adhesion

**Figure 1:**
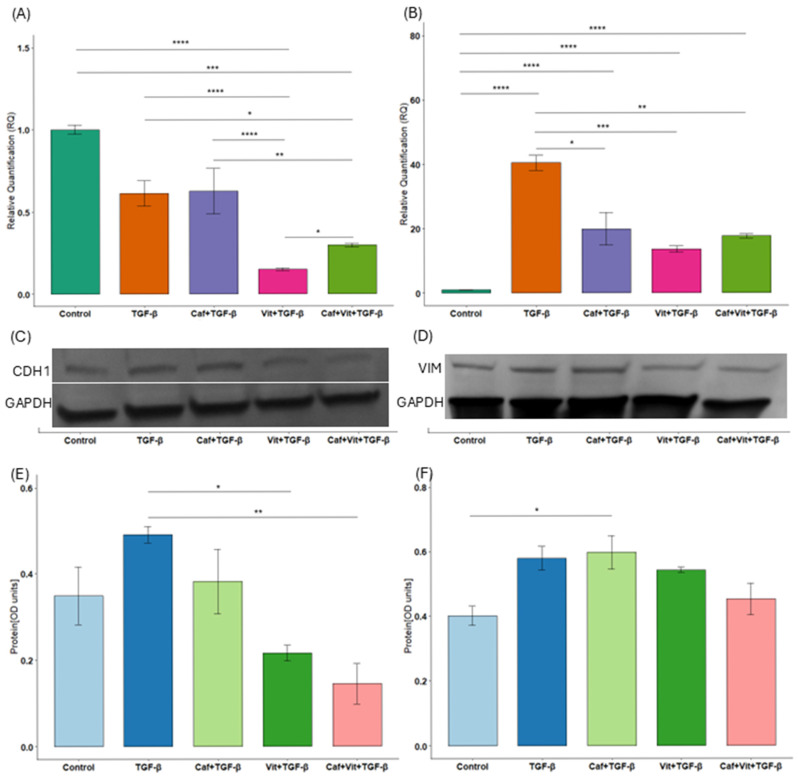
The effect of caffeine and calcitriol on the expression of CDH1 and VIM. (**A**,**B**) mRNA expression and (**C**–**F**) protein level of CDH1 and VIM after exposure to caffeine, calcitriol, and TGF-β. The results are presented in relation to GAPDH as a mean ± SEM (*n* = 3). Original Western blots can be found in Supplementary Materials. * *p* < 0.05, ** *p* < 0.01, *** *p* < 0.001, **** *p* < 0.0001. Caf–caffeine, vit–calcitriol.

We then checked the expression of genes related to cytoarchitecture and cell adhesion, such as CDH1 and VIM (Figure 1A–F). Interestingly, the expression of *CDH1* was significantly decreased after treatment with calcitriol and TGF-β as well as calcitriol, caffeine and TGF-β in comparison to untreated cells (*p* < 0.0001 and *p* = 0.0002, respectively) and cells after TGF-β treatment (*p* < 0.0001 and *p* = 0.01, respectively). Moreover, calcitriol alone, as well as with caffeine, decreased the expression of *CDH1* significantly more than caffeine alone (*p* < 0.0001 and *p* = 0.009, respectively). In contrast, caffeine significantly increased suppressed calcitriol-induced expression (*p* = 0.02). At the protein level, calcitriol alone (*p* = 0.02) and in combination with caffeine (*p* = 0.005) reduced E-cadherin levels compared to treatment with TGF-β. Additionally, the effect size was substantial for mRNA analysis (η^2^ = 0.94, 95%CI [0.84, 1]), and for protein analysis (η^2^ = 0.74, 95%CI [0.33, 1]).

As for the expression of *VIM*, caffeine, calcitriol or calcitriol and caffeine together with TGF-β significantly reduced the expression of this gene in comparison to TGF-β treatment (*p* = 0.02, *p* = 0.0007 and *p* = 0.006 respectively), although the level of this expression remained increased compared to the untreated cells (*p* < 0.0001, *p* < 0.0001 and *p* < 0.0001 respectively). Western blot also showed an increased expression of vimentin by caffeine with TGF- β compared to control cells (*p* = 0.03). Additionally, the effect size was substantial for mRNA analysis (η^2^ = 0.98, 95%CI [0.95, 1]), and for Western blot (η^2^ = 0.66, 95%CI [0.14, 1]).

### 2.2. The Influence of Caffeine and Calcitriol on Expression of Genes Related to Extracellular Matrix Degradation

**Figure 2:**
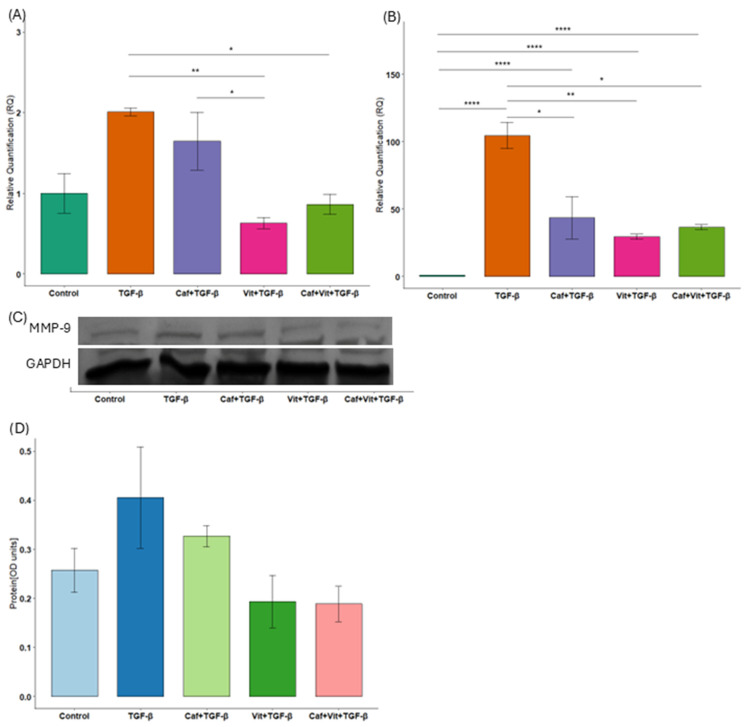
The effect of caffeine and calcitriol on the expression of MMP-2 and MMP-9. (**A**,**B**) mRNA expression of MMP-9 and MMP-2 after exposure to caffeine, calcitriol, and TGF-β. (**C**,**D**) Protein level of MMP-9 after exposure to caffeine, calcitriol, and TGF-β. The results are presented in relation to GAPDH as a mean ± SEM (*n* = 3). Original Western blots can be found in Supplementary Materials. * *p* < 0.05, ** *p* < 0.01, **** *p* < 0.0001. Caf–caffeine, vit–calcitriol.

Next, we examined the expression of genes related to extracellular matrix degradation, such as MMP-2 and MMP-9 (Figure 2A–D). Similar results were obtained for both genes: significantly reduced mRNA expression after treatment with calcitriol and TGF-β as well as calcitriol, caffeine and TGF-β in comparison to TGF-β only (MMP-2: *p* = 0.003 and *p* = 0.01, respectively; MMP-9: *p* = 0.005 and *p* = 0.03, respectively). However, in the case of MMP-2, this expression was nevertheless higher compared to untreated cells (*p* < 0.0001 and *p* < 0.0001, respectively). Moreover, caffeine significantly reduced the expression of *MMP-2* compared to TGF-β (*p* = 0.03) but significantly increased compared to control (*p* < 0.0001). As for *MMP-9* expression, calcitriol significantly decreased expression compared to caffeine (*p* = 0.02). Unfortunately, at the protein level, MMP-9 did not reach statistical significance, but no bands were obtained for MMP-2. Additionally, the effect size was substantial for mRNA analysis (MMP-2: η^2^ = 0.98, 95% CI [0.94, 1]; MMP-9: η^2^ = 0.76, 95% CI [0.37, 1]) and for protein analysis (MMP-9: η^2^ = 0.49, 95% CI [0, 1]).

### 2.3. The Influence of Caffeine and Calcitriol on Vitamin D Receptor (VDR) Expression

**Figure 3:**
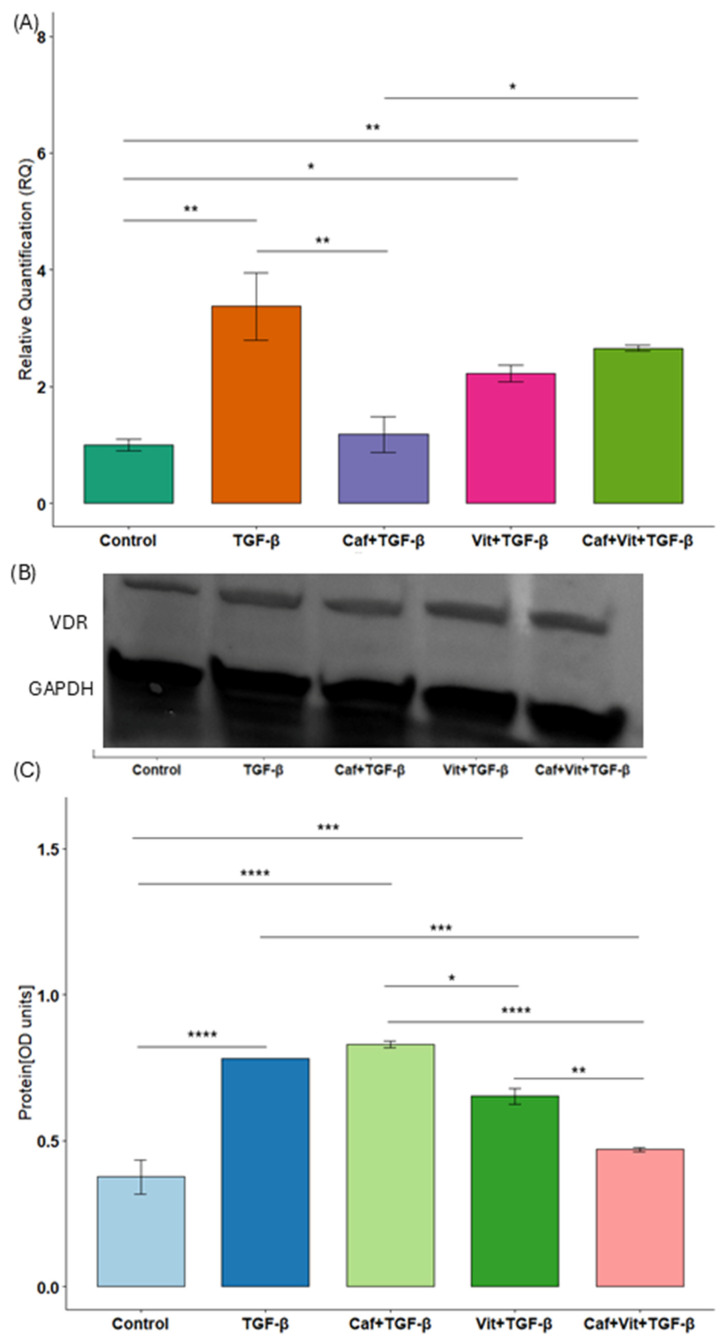
The effect of caffeine and calcitriol on VDR expression. (**A**) mRNA expression and (**B**,**C**) protein level of VDR after exposure to caffeine, calcitriol, and TGF-β. The results are presented in relation to GAPDH as a mean ± SEM (*n* = 3). Original Western blots can be found in Supplementary Materials. * *p* < 0.05, ** *p* < 0.01, *** *p* < 0.001, **** *p* < 0.0001. Caf–caffeine, vit–calcitriol.

We analyzed the expression of VDR to check the effect of caffeine and calcitriol together with TGF-β in Calu-3 cells (Figure 3A–C). Interestingly, the expression of *VDR* was increased after treatment with TGF-β, calcitriol and TGF-β, as well as caffeine, calcitriol and TGF-β in comparison to untreated cells (*p* = 0.001, *p* = 0.02 and *p* = 0.006, respectively). The situation is similar at the protein level: increased VDR levels after TGF-β, caffeine and TGF-β and calcitriol and TGF-β treatments (*p* < 0.0001, *p* < 0.0001 and *p* = 0.0004, respectively).

On the other hand, caffeine inhibited the increased TGF-β-induced mRNA expression (*p* = 0.004). Moreover, calcitriol significantly increased caffeine-induced *VDR* expression (*p* = 0.02), while this effect was reversed at the protein level (*p* < 0.0001). Calcitriol and TGF-β reduced VDR protein levels in comparison to caffeine and TGF-β treatment (*p* = 0.01). Furthermore, caffeine, calcitriol and TGF-β decreased VDR levels in comparison to TGF-β alone as well as calcitriol and TGF-β treatment (*p* = 0.0002 and *p* = 0.009, respectively). Additionally, the effect size was substantial for mRNA analysis (η^2^ = 0.83, 95%CI [0.56, 1]), and for protein analysis (η^2^ = 0.95, 95%CI [0.86, 1]).

## 3. Discussion

In our study, we showed for the first time the effect of caffeine and calcitriol, the active metabolite of vitamin D, on the process related to airway remodeling. We discovered that the examined substances, either separately or in combination, were associated with changes in the expression of genes involved in this process. Furthermore, a potential synergistic effect was demonstrated for *CDH1* at the mRNA level and for the vitamin D receptor at the protein level. In the presented analyses, the η^2^ values were high, suggesting a notable contribution of the factors studied. However, for most proteins, wide confidence intervals suggest greater variability in measurements at the protein level. The opposite pattern was observed only for the vitamin D receptor, as there is a narrower confidence interval in the protein analysis than in the mRNA analysis.

Studies indicate that caffeine may affect airway remodeling by the connective tissue growth factor (CTGF), which is a downstream mediator of TGF-β [15,16,17]. Moreover, caffeine may cause changes in the cytoskeleton by reorganizing major cytoskeletal proteins, including vimentin, in a non-small lung cancer cell line [18]. Immunofluorescence study and Western blot analysis have shown that caffeine can lead to baseline levels of epithelial (ZO-1 and E-cadherin) and mesenchymal (fibronectin and vimentin) markers in a distal renal tubular epithelial cell line [19]. Although our study did not observe a significant effect of caffeine on reduced TGF-β-induced CDH1 expression, we did observe a reduction in *VIM* expression. Unfortunately, caffeine did not inhibit VIM expression to basal levels, as the vimentin level remained statistically higher compared to control cells at the mRNA and protein levels. This may be due to differences in the cell model and experimental conditions. On the other hand, gelatinases, such as MMP-2 and MMP-9, which are members of the metalloproteinase family, are being studied in relation to asthma [20]. Furthermore, increased expression and activity of these gelatinases were observed in patients with chronic obstructive pulmonary disease [21]. Caffeine decreased the expression of proteins and mRNA levels, as well as the activity of MMP-2 and MMP-9 in human acute myeloid leukemic U937 cells [22]. We detected a similar result for *MMP-2* expression after the caffeine treatment. The above results indicate that caffeine can positively influence the epithelial–mesenchymal transition in the airway, induced by TGF-β.

Our study yields heterogenous results regarding the effect of calcitriol on *CDH1* expression. mRNA expression appeared to be more strongly inhibited by calcitriol than by TGF-β or caffeine treatments. Moreover, calcitriol was associated with a decrease in E-cadherin level induced by TGF-β. This is contrary to the findings of other *in vitro* studies. Calcitriol treatment significantly enhanced the mRNA level of *CDH1* in an adherent subpopulation of SW480 cells, which are sensitive to calcitriol [23]. Similar results were obtained at the protein level in SW480 and HT-29 cells [24]. Interestingly, another study using different colon cancer cell lines (SW480, HCT116, and LS174T) found that calcitriol increased *CDH1* expression at the mRNA level. However, it increased CDH1 expression at the protein level only in the SW480 and HCT116 cell lines [25]. Also, another study found that different colorectal cancer cell lines can differ in their expression of EMT, including vimentin and E-cadherin. Cell lines with the most mesenchymal features exhibited high vimentin expression and low E-cadherin expression. Conversely, lines with the fewest mesenchymal features exhibited low or undetectable levels of vimentin and high levels of E-cadherin. Furthermore, using calcitriol on selected lines with varying expression levels revealed that calcitriol has different effects on E-cadherin depending on the cell’s mesenchymal nature. Moreover, different effects of calcitriol on *VIM* expression at the mRNA level were also observed. These results suggest that in some cells, calcitriol is able to increase EMT [26]. Moreover, calcitriol inhibits EMT by suppressing the activation of Smad2/3, STAT3, and β-catenin, according to mechanistic studies in kidney cancer models. This emphasizes that the direction of changes in CDH1 expression may depend on the context of pro-EMT signals and coupling with receptors of such pathways [27]. Nevertheless, in our study, we did not detect an increased expression of *VIM*, despite inhibition of *CDH1* expression after calcitriol and TGF-β treatment in comparison to TGF-β treatment, in contrast to the results of the study conducted on renal cell carcinoma cell lines in which calcitriol increased the E-cadherin level and decreased the vimentin level in TGF-β-treated and TGF-β-untreated cells [27]. Similarly, E-cadherin expression was inhibited by TGF-β on epithelial cells, such as the human bronchial epithelial cell line BEAS-2B. However, it was partially restored by calcitriol when added with TGF-β. E-cadherin expression returned to control levels when calcitriol was administered before and with TGF-β [28]. Moreover, on the same cells, pre-treatment with calcitriol before TGF-β stimulation increased E-cadherin expression and decreased vimentin expression, but only at the protein level. In addition, pre-treatment with calcitriol inhibited TGF-β2-mediated *MMP-2* expression, and TGF-β1-mediated *MMP-9* expression on BEAS-2B cells [29]. Moreover, calcitriol inhibited MMP-2 and MMP-9 protein levels after TNF-α pre-treatment in the nasal polyp-derived fibroblasts, which was confirmed by ELISA and Western blot [30]. Our findings are generally consistent with these results, which demonstrate that calcitriol suppressed the elevated levels of both gelatinases triggered by TGF-β. We detected a synergistic effect between caffeine and calcitriol only in the case of *CDH1* expression: decreased expression against caffeine and increased expression against calcitriol. Nevertheless, caffeine together with calcitriol significantly inhibited TGF-β-induced mRNA expression of *VIM, MMP-2* and *MMP-9,* as well as the E-cadherin protein level.

The inhibition of TGF-β activity by calcitriol appears to be VDR-dependent, as confirmed on BEAS-2B cells with a silenced receptor compared to wild-type cells. Moreover, TGF-β stimulation was associated with an increase in the expression of VDR on protein as well as mRNA levels [28]. Similarly, in human pancreatic cancer cells, TGF-β also increased *VDR* expression. However, adding calcitriol to TGF-β treatment did not significantly reduce the *VDR* mRNA level [31]. This phenomenon was also demonstrated in our study on mRNA and protein levels. Interestingly, a study by Fiz et al. [31] also shows a reduction in *CDH1* expression under the influence of TGF-β. Although in contrast to our results, the addition of calcitriol slightly increased this level, but the result is not statistically significant. In the case of caffeine, we detected for the first time its ability to reduce TGF-β-induced *VDR* expression. In contrast, the addition of calcitriol to caffeine significantly increased this mRNA expression, as compared to caffeine, and as compared to untreated cells, although this increased mRNA expression was not significantly higher compared to calcitriol treatment. Interestingly, a different result was obtained at the protein level. The combination of calcitriol and caffeine was associated with reduction in the level of vitamin D receptors in samples treated separately with calcitriol, caffeine, and only TGF- β. In line with these observations, Rapuri et al. [32] reported a dose-dependent reduction in calcitriol-induced VDR expression that was observed in osteoblast-like cells.

This study has several limitations. First, this was a pilot *in vitro* study, which limits the extent to which the findings can be extrapolated to *in vivo* conditions. Second, the analysis was performed on a single cell line, and for some proteins, such as MMP-2, detectable bands were not observed, which may raise technical concerns. At the same time, the absence of MMP-2 signaling may also reflect a real biological effect or context-dependent regulation of its expression in this model, which requires further clarification. Third, several of the observed changes were not statistically significant, which limits the strength of the conclusions and suggests that biological variability or insufficient statistical power may have influenced the outcomes. Finally, mechanistic explanations for the observed effects were not investigated and functional studies were not conducted, as the main objective of this study was to provide preliminary insights that may guide future, more comprehensive studies. Moreover, discrepancies were observed between mRNA and protein expression, likely reflecting post-transcriptional regulation, protein stability, or technical variability.

Calu-3 cells originate from a human lung adenocarcinoma [33]; however, under standard culture conditions, they retain a well-differentiated epithelial phenotype, characterized by high expression of tight-junction proteins, E-cadherin, and the ability to form polarized monolayers with high transepithelial electrical resistance [34,35]. These properties have led to the widespread use of Calu-3 cells as a functional model of the human airway epithelial barrier *in vitro* [36]. EMT is seen as a dynamic and context-dependent process rather than a binary switch, which is particularly relevant in cancer-derived models [37].

Future studies should investigate how the observed anti-EMT-related effects of calcitriol and caffeine may translate into broader regulation of airway remodeling, including epithelial integrity, fibroblast activity, and extracellular matrix dynamics. Further studies, including dose–response analyses and combination approaches, may help clarify whether these agents could be used to develop targeted strategies that counteract airway structural changes in chronic respiratory diseases.

In summary, the effects of calcitriol and caffeine on EMT-related pathways in this model were modest and inconsistent, highlighting the need for further studies involving additional cell lines and broader validation at the protein level.

## 4. Materials and Methods

### 4.1. Materials

Calu-3 (human lung adenocarcinoma cell line) was obtained from ATCC (Manassas, VA, USA). McCoy’s 5A medium, 1,25(OH)_2_D_3_, Penicillin–Streptomycin solution, caffeine, goat anti-mouse IGG, goat anti-rabbit IGG, protease inhibitor cocktail, and RIPA buffer were purchased from Merck (Darmstadt, Germany). Fetal bovine serum (FBS) was purchased from Genos (Lodz, Poland). Primary antibodies, such as E-cadherin (sc-8426), GAPDH (sc-47724), MMP-9 (sc-10737), Vimentin (sc-6260), MMP-2 (sc-13594), and VDR (sc-13133), were purchased from Santa Cruz Biotechnology, Inc. (Dallas, TX, USA). Total RNA Mini kit was purchased form A&A Biotechnology (Gdansk, Poland). TGFB1 Recombinant Human Protein, High-Capacity cDNA Reverse Transcription Kit, TaqMan^TM^ Gene Expression Master Mix, TaqMan™ gene expression assays: MMP-9 (Hs009557562_m1), MMP-2 (Hs01548727_m1), VIM (Hs00958111_m1), CDH1 (Hs01023895_m1), GAPDH (Hs02786624_g1), VDR (Hs01045843_m1) were purchased from Thermo Fisher Scientific (Waltham, MA, USA). ExpressPlus PAGE gel of 4–20% and MOPS Running buffer powder were obtained from GenSignal (Poznan, Poland).

### 4.2. Cell Culture and Treatments

Calu-3 cells were cultured in McCoy’s 5A medium supplemented with 10% FBS and penicillin–streptomycin solution and maintained at 5% CO_2_. A total of 24 h before experiments, when the cells reached 80–90% confluence, the medium was changed to free FBS. Cells were treated with 10 ng/mL of TGF-β for 24 h (TGF-β); with 5 mM of caffeine and 10 ng/mL of TGF-β for 24 h (Caf + TGF-β); with 100 nM of 1,25(OH)_2_D_3_ for 24 h and 10 ng/mL of TGF-β for the next 24 h (Vit + TGF-β); and with 100 nM of 1,25(OH)_2_D_3_ for 24 h, 5 mM of caffeine and 10 ng/mL of TGF-β for the next 24 h (Caf + Vit + TGF-β). For the control condition, cells were incubated in FBS-free medium for 72 h.

### 4.3. Analysis of Gene Expression

Total mRNA was isolated using the Total RNA Mini kit and subsequently reverse transcribed with the High-Capacity cDNA Reverse Transcription Kit, following the manufacturer’s instructions. Gene expression analysis of CDH1, MMP-9, MMP-2, ACTA2, VIM and VDR was assessed using the TaqMan^TM^ Gene Expression Master Mix with TaqMan™ gene expression assays in triplicate according to the manufacturer’s protocols. The 2^−ΔΔCt^ method was used to calculate gene expression. Statistical analyses were performed on ΔCt values. For graphical presentation, results were transformed to relative quantities (RQ), and the standard error of the mean was calculated using error propagation. GAPDH was used as the reference gene for normalization.

### 4.4. Immunoblotting

Total protein was isolated using RIPA buffer containing a protease inhibitor cocktail. The protein was then separated using 4–20% ExpressPlus PAGE gel for 60 min (110 mA). After this, the protein was transferred into a nitrocellulose membrane using eBlot Protein Transfer System (Genscript, Piscataway, NJ, USA). The membrane was blocked for 1 h using 5% non-fat milk dissolved in TBST buffer. Next, the membrane was incubated with primary antibodies with 1% non-fat milk dissolved in TBST buffer at 4 °C overnight and, next, with secondary antibodies for 90 min at room temperature. Results were visualized using BCIP/NBT alkaline phosphatase substrate. Densitometric image analysis was performed in Image J 1.54g software (Wayne Rasband, National Institutes of Health, Bethesda, Washington, MD, USA).

### 4.5. Statistical Analysis

Statistical analysis of the data was performed in R (version 4.2.2). The Kolmogorov–Smirnov test was used to check the distribution of data. If the data had normal distribution, Levene’s test was used to check the equality of variances. One-way analysis of variance (ANOVA) followed by Tukey’s honestly significant difference (HSD) post hoc test was used to determine differences in several groups. Eta squared (η^2^) with a 95% confidence interval was calculated to measure the effect size in ANOVA. Results were considered statistically significant at *p* < 0.05.

## 5. Conclusions

This study assessed the effect of vitamin D and caffeine on processes related to airway remodeling. Changes in transcriptional levels of selected markers associated with airway remodeling were observed, while protein-level analysis was limited. This suggests a potential modulatory effect of vitamin D and caffeine against processes leading to pathological remodeling. Preliminary results indicate a possible role of these substances as adjunctive agents in future therapeutic strategies targeting obstructive diseases; however, since this was an *in vitro* study, the conclusions should be interpreted with caution and require confirmation in further *in vivo* research.

## Data Availability

The original contributions presented in this study are included in the article/Supplementary Material. Further inquiries can be directed to the corresponding author.

## Supplementary Materials

The following supporting information can be downloaded at: https://www.mdpi.com/article/10.3390/ijms27073087/s1.
