# Supplementary material for: Modulators of Airway Remodeling: The Role of Caffeine and Calcitriol
Source: Int J Mol Sci. 2026 Mar 28;27(7):3087. doi: 10.3390/ijms27073087 (PMC13074156; doi:10.3390/ijms27073087)
Supplement: Supplementary file 1 [file ijms-27-03087-s001.zip › ijms-4174620-supplementary.pdf]

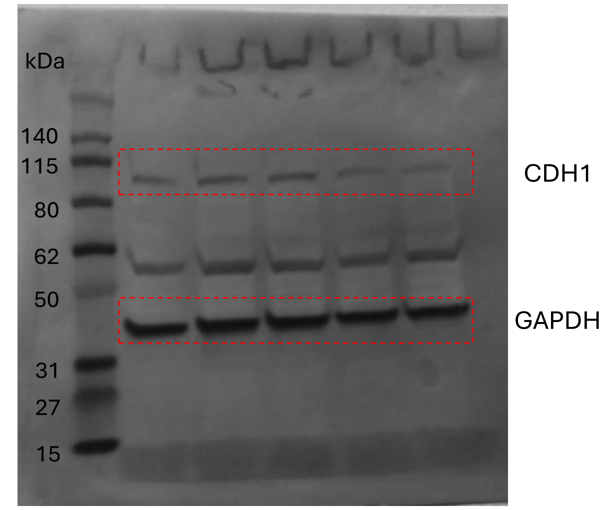

Original blots corresponding to Figure 1C.

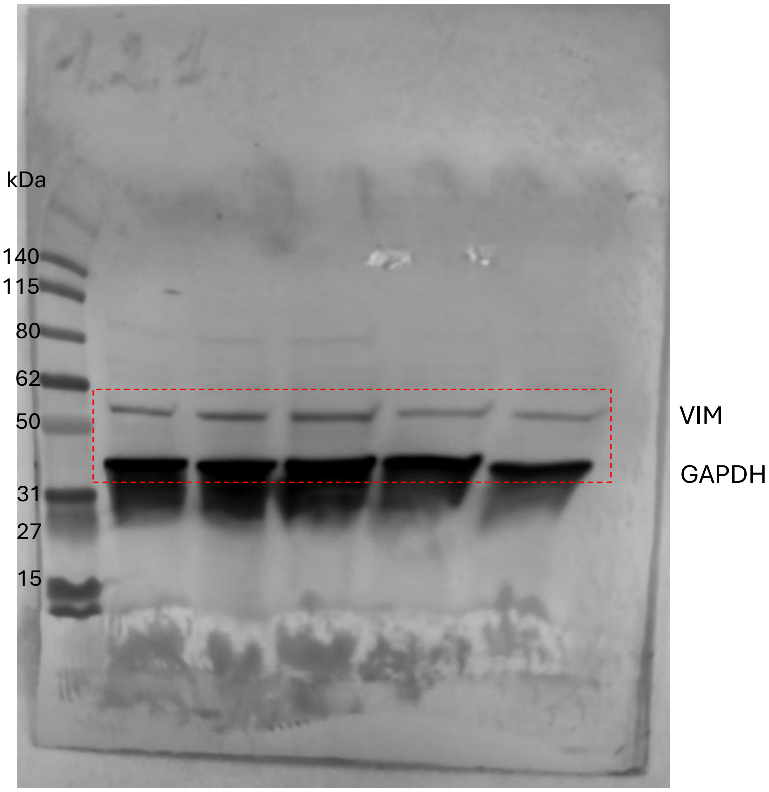

Original blots corresponding to Figure 1D.

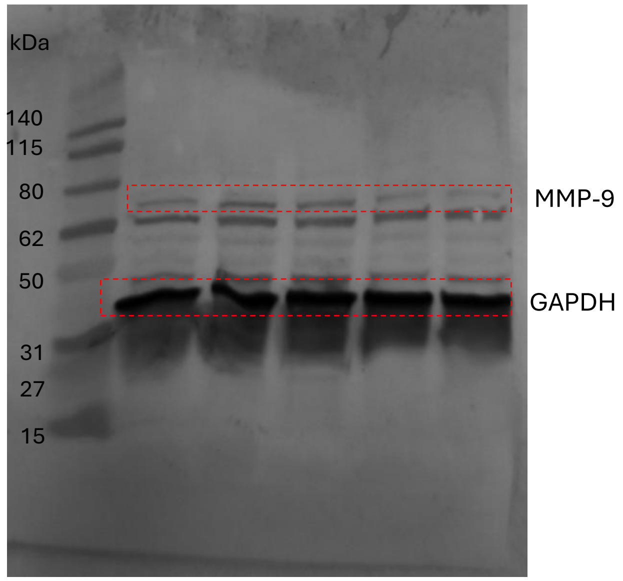

Original blots corresponding to Figure 2C.

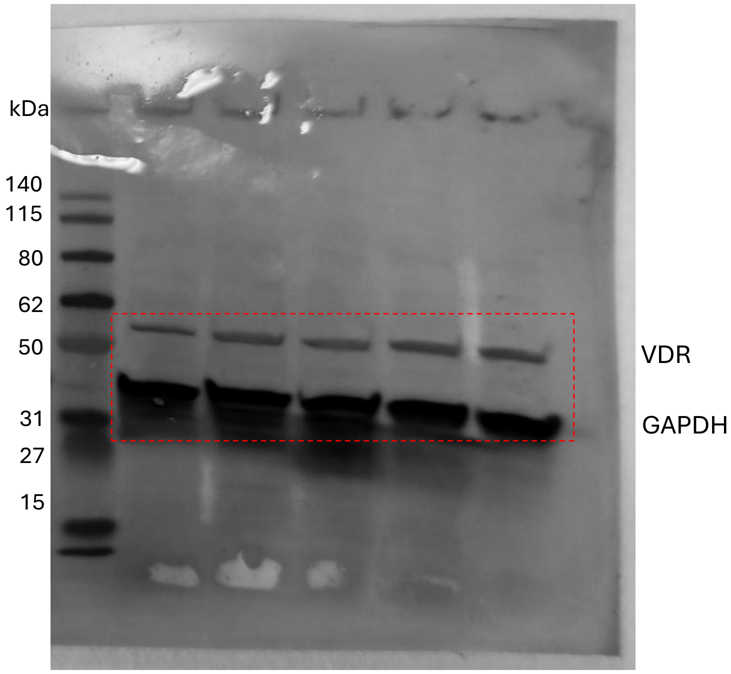

Original blots corresponding to Figure 3B.
